# Modulation of Vasomotive Activity in Rabbit External Ophthalmic Artery by Neuropeptides

**DOI:** 10.1155/2012/498565

**Published:** 2012-02-09

**Authors:** Esmeralda Sofia Costa Delgado, Carlos Marques-Neves, Maria Isabel Sousa Rocha, José Paulo Pacheco Sales-Luís, Luís Filipe Silva-Carvalho

**Affiliations:** ^1^Clinical Department, CIISA, Faculty of Veterinary Medicine, Technical University of Lisbon, Alameda da Universidade Técnica, 1300-477 Lisbon, Portugal; ^2^Instituto de Medicina Molecular and Instituto de Fisiologia, Faculdade de Medicina, Universidade de Lisboa, Avenida Professor Egas Moniz, 1649-028 Lisbon, Portugal

## Abstract

*Purpose*. To investigate the vasomotive activity upon the external ophthalmic artery of vasointestinal peptide (VIP) and neuropeptide Y (NPY) using a previously developed model. *Methods*. Isolated rabbit eyes (*n* = 12) were perfused *in situ* with tyrode through the external ophthalmic artery. Effects of intra-arterial injections of NPY 200 *μ*g/ml (Group A; *n* = 6) and VIP 200 *μ*g/ml (Group B; *n* = 6) on the recorded pressure were obtained. For statistical analysis, Student's paired *t*-test and Fast Fourier Transform were used. *Results*. Spontaneous oscillations were observed before any drug administration in the 12 rabbit models. NPY produced an increase in total vascular resistance and a higher frequency and amplitude of oscillations, while VIP evoked the opposite effects. *Conclusions*. This study provides evidence of vasomotion in basal conditions in rabbit external ophthalmic artery. Concerning drug effects, NPY increased arterial resistance and enhanced vasomotion while VIP produced opposite effects which demonstrates their profound influence in arterial vasomotion.

## 1. Introduction

Disturbances of ocular blood flow are involved in many ophthalmic diseases and therefore are of the utmost clinical relevance. The eye is one of the best perfused organs in the body. In humans and in experimental animals, the eye has two separate systems of blood vessels, which anatomically and physiologically differ: the retinal vessels, which supply part of the retina, and the uveal or ciliary blood vessels, which supply the rest of the eye. There is autonomic innervation of the extraocular vessels as well as of choroidal vessels [1, 2]. In primates, innervation of the central artery occurs as far as to the lamina cribrosa. There is no innervation of vessels of the retina although alpha, beta-adrenergic and cholinergic receptors are present [3, 4]. There are studies describing some innervation of the optic nerve head [5].

Besides there is mounting evidence that the retinal blood vessels have receptors for and sensitivity to neurotransmitters and neurohormones, including biologically active peptides [6, 7]. NPY and VIP are two neuropeptides that occur in peripheral nerves supplying both the eye and blood vessels to the brain [7, 8].

Vasoactive intestinal peptide (VIP) is a peptide hormone containing 28 amino acid residues produced in many areas of the human body including the gut, pancreas, and suprachiasmatic nuclei of the hypothalamus in the brain and is neuroprotective [9].

Neuropeptide Y (NPY) is a 36-amino acid peptide neurotransmitter found in the brain and autonomous nervous system, secreted by the hypothalamus, and it has been associated with several physiologic processes in the brain, blocking nociceptive signals and augmenting the vasoconstrictor effects of noradrenergic neurons [10].

They are both vasoactive, VIP nerve fibers are parasympathetic causing vasodilation, and NPY is generally associated with the sympathetics, although it is also found in some parasympathetic neurons supplying the eye, suggesting a mixed autonomic origin [7]. In several organs, VIP seems to be colocalized with neuronal NOS and the same holds true for nitrergic choroidal innervation [11].

In this work, we used an isolated model of rabbit eye previously developed [12] to study the evoked vasomotor responses with NPY and VIP intra-arterial administration upon perfusion pressure and periodic oscillations at the rabbit external ophthalmic artery *in vitro*.

## 2. Methods

### 2.1. Anaesthesia and Surgical Procedures

Twelve hybrid NewZealand rabbits of either sex were used, weighing between 2,10 Kg and 3,90 Kg, mean 3,20 ± 0.157 Kg, anaesthetized with pentobarbital sodium (40 mg/Kg body weight iv, Eutasil, Sanofi, Portugal), supplemented as necessary, tracheostomised and artificially ventilated with O_2_-enriched air applied using a positive pressure ventilator (Harvard Apparatus Ltd, UK). Body temperature was monitored and maintained constant (38-39°C) by using a servocontrolled heating blanket (Harvard Apparatus Ltd, UK). The external carotid artery and jugular vein were cannulated for the administration of drugs and bleeding, respectively. During the surgical procedures, arterial pressure (Neurolog System, Digitimer, UK), respiratory rate, and ECG (Neurolog System, Digitimer, UK) were monitored to assess the depth of anaesthesia. Heparin (1000 UI/Kg) was perfused through the external carotid artery, and a waiting time of 20 min was respected. After that euthanasia was performed with an overdose of pentobarbital sodium, the head was sectioned at cervical level and perfusion was commenced at the external carotid artery. Encephalon and intact cranial nerves were mechanically destroyed using a scalpel blade that entered the cranium through the foramen magnum, to ensure that the interference of stimuli originating outside the ocular globe in the central nervous system and the autonomous nervous system was abolished. The protocols and procedures were approved by the University Ethics Committee and conformed to the Helsinki Declaration.

### 2.2. Isolated Eye Model

External ophthalmic arteries were exposed, and a 0,6 mm outside diameter polypropylene tube was placed in the vessels with the aid of a surgical microscope (Shin Nippon, Japan). The three-way catheter was further connected to a continuous intravenous infusion apparatus (Semat, series 81706, UK) and to a pressure transducer (Neurolog System, Digitimer, UK). The effect of intraluminal pressure as a measure of total vascular resistance was assessed. The head was maintained immerse in a glass chamber containing O_2_-enriched tyrode at a constant temperature of 38°C and controlled pH. Continuous infusion was commenced at a flow rate of 135 *μ*L/min.

There was a period between the death of the animal and the beginning of the experiment that corresponded to 40.6 ± 0.93 minutes in Group A and 39.4 ± 0.93 minutes in Group B (*n* = 12, *P* = 0.387). Once perfusion commenced, an equilibration period was respected that corresponded to 16.0 ± 0.71 minutes in Group A and 15.4 ± 0.51 minutes in Group B (*n* = 12, *P* = 0.511). On what concerns the total time of an experiment, from the euthanasia of the animal until the end of the experiment, it was 110.4 ± 2.42 minutes in Group A and 109,2 ± 2,13 minutes in Group B (*n* = 12, *P* = 0.719). Since *P* values were greater than 0.05, differences were considered not significant, that is, the difference between means is not significantly greater than expected by chance.

### 2.3. Drug Injections

In group A (*n* = 6), response curves to three injections of NPY in a concentration of 200 *μ*g/mL [13] were obtained. In group B (*n* = 6), response curves to three injections of VIP [13] in a concentration of 200 *μ*g/mL were obtained. Injections were given intra-arterially in a volume of 0.1 mL in a system derivation, with a 20-minute interval and the waiting time respected intended to allow the pressure to return to a stable baseline. In the end, the pressure was calibrated to a value of 40 mm Hg to adjust pressure values obtained. Finally, methylene blue dye was injected through the ophthalmic artery to confirm eye perfusion by blue staining.

### 2.4. Drugs and Solutions

Tyrode solution comprises NaCl (137 mM), KCl (2.7 mM), CaCl_2_ (1.8 mM), MgCl_2_ (0.49 mM), NaH_2_PO_4_ (0.36 mM), NaHCO_3_ (11.9 mM), glucose (5.6 mM), and distilled water (1 L). NPY (N9409) and VIP (V6130) both from Sigma Aldrich Chemie Gmbh P. O. 1120, 89552 Steinheim, Germany, were diluted in distilled water and freshly prepared.

### 2.5. Data Analysis

For the variables recorded, the baseline values were taken immediately before the beginning of the injection and compared with the ones obtained in the peak of the response. Perfusion pressure, frequency, and amplitude of the oscillations were evaluated before and after the drug injections. All recorded variables were digitised (Instrutech VR100B, Digitimer Ltd, UK) and recorded on videotape. Off-line analysis was done using a computer A/D system with data capture and analysis software (Chart for Windows, 5.0, USA). Data were analysed with GraphPad (InStat, version 3.00 for Windows 95, GraphPad Software, San Diego California USA) and are reported as mean ± standard error. Student's *t*-test for paired comparisons were performed to compare values obtained before and after the drug injections for perfusion pressure, frequency, and amplitude of the oscillations with *P* < 0.05 testing for significance. Data were tested for normal distribution with the Kolmogoroff-Smirnoff test.

Episodes of vasomotion were recorded, and the amplitudes and frequencies of the oscillations were characterized by fast fourier analysis. The frequency spectra of the oscillations are expressed in Hertz (Hz).

## 3. Results

### 3.1. Spontaneous Oscillations

**Figure 1:**
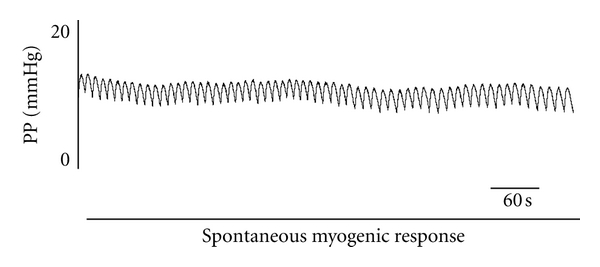
Example of basal periodic oscillations in vascular tone observed before any drug administration. On the *x* axis, the perfusion pressure recorded at the rabbit external ophthalmic artery is represented in mm Hg. In the *y* axis, we represent the time in seconds.

Without any drug administration, spontaneous myogenic responses were observed in the 12 rabbit models showing oscillations of a medium frequency of 7.7 ± 1.71 oscillations per minute and a medium amplitude value of 2.1 ± 1.13 mm Hg (Figure 1).

### 3.2. NPY and VIP Effects on Perfusion Pressure

In Group A (*n* = 6) of experiments we observed that the intra-arterial delivered NPY elicited vasoconstriction. The first intra-arterial injection of 0.1 mL of NPY 200 *μ*g/mL evoked an increase of 41%, the second intra-arterial injection elicited an increase of 25%, and the third registered an increase of 35% (Table 1). In Group B (*n* = 6), the arteries dilated in response to VIP. With the first intra-arterial injection, we registered a decrease of 29%, with the second administration a decrease of 29%, and the third evoked a decrease of 27% (Table 2).

### 3.3. NPY and VIP Effects on the Frequency of Periodic Oscillations

In Group A, with NPY, the frequency of the oscillations increased. The first injection resulted in increase of 36%, the second produced an increase of 47%, and the third an increase of 40% (Table 1). In Group B, with VIP, the frequency of the oscillations decreased. With the first intra-arterial injection, we registered a frequency decrease of 35%, with the second a decrease of 46%, and with the third administration a decrease of 45% (Table 2).

### 3.4. NPY and VIP Effects on the Amplitude of Periodic Oscillations

**Figure 2:**
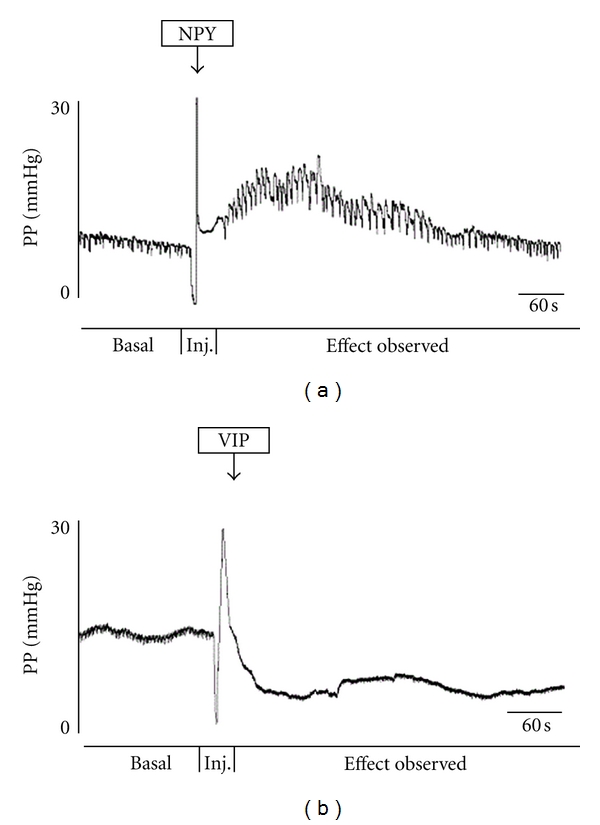
Example of changes in vascular tone observed after an intra-arterial injection of NPY in group A, causing vasoconstriction and increase in frequency and amplitude of the oscillations. We can see an example of an effect of an intra-arterial injection of VIP in perfusion pressure, producing vasodilation and decrease in frequency and amplitude of the oscillations. On the *x* axis, the perfusion pressure recorded at the rabbit external ophthalmic artery is represented in mm Hg. In the *y* axis, we represent the time in seconds.

In Group A, with NPY, the amplitude of the oscillations increased. With the first injection, we registered an increase of 48%, with the second an increase of 63% and with the third an increase of 57% (Table 1). In Group B there was a decrease in the amplitude of the oscillations. The first injection of VIP made the amplitude of the oscillations decrease 30%, the second elicited a decrease of 29%, and the third a decrease of 25% (*P* = 0.004) (Table 2, Figure 2).

### 3.5. Fast Fourier Analysis

**Figure 3:**
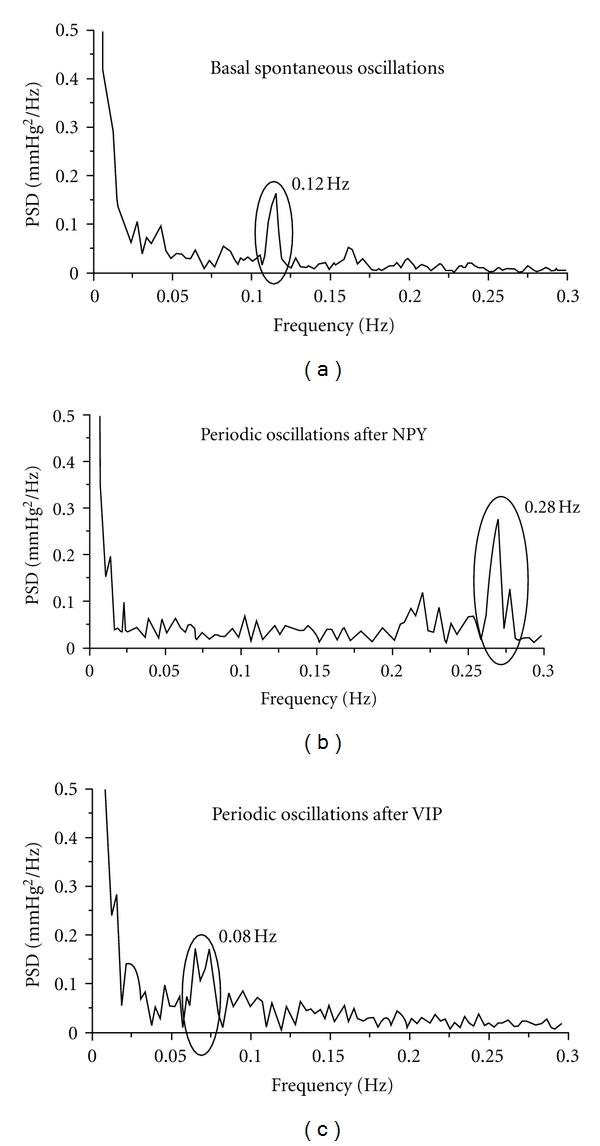
In the upper panel, the power spectrum density (PSD) of basal spontaneous oscillations obtained as a result of mathematical analysis with the Fast Fourier algorithm shows a major band at 0.12 Hz. In the middle panel, the power spectrum density obtained after Fourier analysis of the oscillations after NPY injection shows a major band around 0.28 Hz which results in an increase in the frequency of the oscillations, so there was a marked increase in the frequency of the spontaneous oscillations. In the lower panel, the power spectrum density obtained after VIP injection shows a peak frequency of 0.08 Hz, so there was a decrease in the frequency of the spontaneous oscillations.

As a result of the mathematical analysis with Fast Fourier Transform, a power spectrum density (PSD) was built for each rabbit. The most prominent frequency was considered as the principal frequency and was used to characterize the vasomotion pattern. The frequency spectrums illustrated the results displayed in Figure 3, although we could see the presence of several superimposed frequencies. So there was a marked increase in the frequency of the oscillations with NPY and a decrease following VIP administration (Figure 3).

## 4. Discussion

In this study, we characterized the reactivity of rabbit ocular vasculature to intraluminal pressure by perfusing the feeding external ophthalmic artery in a head-mounted preparation. This, thus, ruled out a central nervous system (CNS) mediation and led the authors to postulate that this *in vitro* model abolished the interference of stimuli originating outside the ocular globe, through the CNS, therefore proved that the spontaneous oscillations observed in basal conditions were independent of central regulation, being interpreted as vasomotion [12].

However, one cannot interpret this ex vivo model as representing a purely vasomotor paradigm devoid of any autonomic innervation. There are some 20 intrinsic choroidal neurons in the rabbit eye [11], and the sphenopalatine ganglion still intact in this preparation may receive trigeminal peptidergic collaterals also in the rabbit as it does in the rat. The brain vasculature was shown to be one target structure for the innervated principal cells in the sphenopalatine ganglion [14], so ocular vasculature could be another target. Thus, peripheral “pre-central” neuronal reflexes may superimpose on vasomotor activity in the ocular vasculature.

Vasomotion is periodic oscillations in the tone of arterioles resulting in an intermittent supply of blood to individual microcirculatory units, facilitating oxygenation of tissues near prevenular capillaries [15]. The phenomenon is therefore assumed to play an important role in microcirculation. In retinal microcirculation [16] and in isolated bovine [17] and porcine [18] retinal arterioles, the presence of vasomotion has been demonstrated. Recently, it has been proposed that disturbances in vasomotion might be important factors in the development of retinal lesions in diabetic maculopathy [19]. Therefore, a further characterization of vasomotion in ocular arteries is pertinent.

Although, in our study, the experimental design cannot determine the arterial segment responsible for the vasomotion observed, external ophthalmic artery/choroid/retina, clinical studies use ophthalmic artery haemodynamics as a measure of the overall function of the ophthalmic circulation [20].

We also quantified the responses to NPY and VIP, two neuropeptides that occur in peripheral nerves supplying the eye [7], by monitoring the perfusion pressure near the entry point to the eye as a measure of total vascular resistance.

Concerning the effects on vasoreactivity of intra-arterial administration, NPY and VIP showed opposite effects: NPY elicited a vasoconstrictor response, and VIP produced vasodilation of the external ophthalmic artery and its collaterals. These results show that, under *in vitro* perfusion, eye arteries present similar responses to NPY and VIP than those observed in *in vivo* models [21].

Yet previous studies have shown that VIP receptors and VIP-containing neurons are not uniformly distributed in the arterial vasculature and that VIP may have selective vasodilatory effects [22].

On what regards the effects on vasomotion, NPY produced an increase in total vascular resistance and vasomotion became more evident, exhibiting a higher frequency and amplitude of oscillations. The evoked vasomotor responses with VIP were vasodilation and decrease of the frequency and amplitude of the oscillations of myogenic tone, which is in favour of a functional role of perivascular peptides in the control of ocular circulation.

Being so, the results of this investigation have shown that neuropeptidergic innervation of the rabbit eye has a profound influence in arterial vasomotion, which might be important in diagnosis or therapeutics of ocular ischemic diseases.

## Figures and Tables

**Table 1:** PP: Perfusion Pressure (mm Hg); Freq: Frequency of oscillations (number oscillations/min); Ampl: Amplitude of oscillations (mm Hg); NPY = Neuropeptide Y.

Group A									
	Basal	First NPY	*P*	Basal	Second NPY	*P*	Basal	Third NPY	*P*
PP	9.1 ± 1.67	19.2 ± 3.49	<0.015	11.9 ± 1.66	15.9 ± 2.49	<0.008	10.7 ± 1.29	16.5 ± 2.23	<0.015
Freq	10.4 ± 5.00	19.2 ± 9.27	<0.002	7.2 ± 4.35	17.7 ± 8.83	0.034	5.7 ± 3.54	13.5 ± 7.38	0.011
Ampl	1.4 ± 0.73	2.7 ± 1.17	<0.021	0.7 ± 0.26	1.9 ± 0.71	<0.01	0.3 ± 0.06	0.7 ± 0.12	<0.01

**Table 2:** PP: Perfusion Pressure (mm Hg); Freq: Frequency of oscillations (number oscillations/min); Ampl: Amplitude of oscillations (mm Hg); VIP: Vasointestinal Peptide.

Group B									
	Basal	First VIP	*P*	Basal	Second VIP	*P*	Basal	Third VIP	*P*
PP	23.9 ± 6.98	15.4 ± 4.31	<0.006	20.8 ± 5.40	12.9 ± 3.14	<0.004	18.6 ± 4.32	12.2 ± 3.37	0.001
Freq	13.6 ± 7.14	8.3 ± 5.00	0.001	9.8 ± 5.18	5.9 ± 3.80	0.015	7.8 ± 4.06	3.7 ± 2.16	0.004
Amp	2.7 ± 1.53	1.9 ± 1.17	0.050	2.4 ± 1.39	1.7 ± 1.11	<0.032	2.0 ± 1.23	1.5 ± 1.13	0.004

## References

[1] Bill A, Sperber GO (1990). Control of retinal and choroidal blood flow. *Eye*.

[2] Kiel JW (1994). Choroidal myogenic autoregulation and intraocular pressure. *Experimental Eye Research*.

[3] Mann RM, Riva CE, Stone RA, Barnes GE, Cranstoun SD (1995). Nitric oxide and choroidal blood flow regulation. *Investigative Ophthalmology and Visual Science*.

[4] Steinle JJ, Krizsan-Agbas D, Smith PG (2000). Regional regulation of choroidal blood flow by autonomic innervation in the rat. *American Journal of Physiology*.

[5] Bergua A, Schrödl F, Neuhuber WL (2003). Vasoactive intestinal and calcitonin gene-related peptides, tyrosine hydroxylase and nitrergic markers in the innervation of the rat central retinal artery. *Experimental Eye Research*.

[6] Hoste AM, Boels PJ, Brutsaert DL, De Laey JJ (1989). Effect of alpha-1 and beta agonists on contraction of bovine retinal resistance arteries *in vitro*. *Investigative Ophthalmology and Visual Science*.

[7] Ye X, Laties AM, Stone RA (1990). Peptidergic innervation of the retinal vasculature and optic nerve head. *Investigative Ophthalmology and Visual Science*.

[8] Edvinsson L (1985). Functional role of perivascular peptides in the control of cerebral circulation. *Trends in Neurosciences*.

[9] Fahrenkrug J, Emson PC (1982). Vasoactive intestinal polypeptide: functional aspects. *British Medical Bulletin*.

[10] Colmers WF, El Bahn B (2003). Neuropeptide Y and Epilepsy. *Epilepsy Currents/American Epilepsy Society*.

[11] Flügel C, Tamm ER, Mayer B, Lutjen-Drecoll E (1994). Species differences in choroidal vasodilative innervation: evidence for specific intrinsic nitrergic and VIP-positive neurons in the human eye. *Investigative Ophthalmology and Visual Science*.

[12] Delgado E, Marques-Neves C, Rocha I, Sales-Luís J, Silva-Carvalho L (2009). Intrinsic vasomotricity and adrenergic effects in a model of isolated rabbit eye. *Acta Ophthalmologica*.

[13] Yao W, Sheikh SP, Ottesen B, Jorgensen JC (1996). The effect of neuropeptides on vessel tone and cAMP production. *Annals of the New York Academy of Sciences*.

[14] Suzuki N, Hardebo JE, Owman C (1989). Trigeminal fibre collaterals storing substance P and calcitonin gene-related peptide associate with ganglion cells containing choline acetyltransferase and vasoactive intestinal polypeptide in the sphenopalatine ganglion of the rat. An axon reflex modulating parasympathetic ganglionic activity?. *Neuroscience*.

[15] Tsai AG, Intaglietta M (1993). Evidence of flowmotion induced changes in local tissue oxygenation. *International Journal of Microcirculation*.

[16] Braun RD, Linsenmeier RA, Yancey CM (1992). Spontaneous fluctuations in oxygen tension in the cat retina. *Microvascular Research*.

[17] Delaey C, Van de Voorde J (2000). Pressure-induced myogenic responses in isolated bovine retinal arteries. *Investigative Ophthalmology and Visual Science*.

[18] Jeppesen P, Aalkjær C, Bek T (2003). Myogenic response in isolated porcine retinal arterioles. *Current Eye Research*.

[19] Bek T (1999). Diabetic maculopathy caused by disturbances in retinal vasomotion. A new hypothesis. *Acta Ophthalmologica Scandinavica*.

[20] Yu DY, Su EN, Cringle SJ, Yu PK (2003). Isolated preparations of ocular vasculature and their applications in ophthalmic research. *Progress in Retinal and Eye Research*.

[21] Lütjen-Drecoll E (2006). Choroidal innervation in primate eyes. *Experimental Eye Research*.

[22] Sidawy AN, Sayadi H, Harmon JW (1989). Distribution of vasoactive intestinal peptide and its receptors in the arteries of the rabbit. *Journal of Surgical Research*.

